# Liposomal Nanosystems Versus Hydrogels in the Prevention and Treatment of Metabolic Diseases

**DOI:** 10.3390/gels11110917

**Published:** 2025-11-16

**Authors:** Mihaela-Carmen Eremia, Ramona-Daniela Pavaloiu, Fawzia Sha’at, Dana Maria Miu, Gabriela Savoiu, Anca Daniela Raiciu

**Affiliations:** 1 National Institute for Chemical-Pharmaceutical Research & Development—ICCF, Bucharest, 112 Vitan Avenue, 3rd District, 031299 Bucharest, Romania; pavaloiu.daniella@gmail.com (R.-D.P.); fawzya.shaat@gmail.com (F.S.); dana.miu92@gmail.com (D.M.M.); gabriela.savoiu@gmail.com (G.S.); 2 Department of Pharmacognosy, Faculty of Pharmacy, University of Titu Maiorescu, Phytochemistry, Phytotherapy, 16 Gh. Sincai Street, 040441 Bucharest, Romania; daniela_raiciu@yahoo.com

**Keywords:** liposomes, hydrogels, metabolic diseases, delivery systems

## Abstract

Liposomal nano-systems and hydrogels are two types of nano-technological systems that have promising applications in the prevention and treatment of metabolic diseases (diabetes, obesity, dyslipidemias, and metabolic syndrome), which are a major public health concern worldwide. Advances in nanotechnology and biomaterials have enabled the development of new platforms for the controlled delivery of nutrients or bioactive compounds in order to solve these issues. This review compares the characteristics, advantages, and limitations of these two systems, with a focus on their applicability in the prevention and treatment of metabolic diseases.

## 1. Introduction

Obesity, type 2 diabetes, vitamin deficiencies, and metabolic syndrome are some of the many chronic conditions that affect millions of people worldwide, placing significant strain on healthcare systems and requiring effective and safe therapeutic approaches. Thus, nutritional strategies aimed at improving metabolic balance have attracted increasing interest. Advanced nutrient delivery systems can improve the bioavailability, stability, and therapeutic efficacy of administered substances [1,2,3].

Pharmaceutical nanotechnology is an innovative field in the development of modern drug delivery systems that aim to enhance therapeutic efficacy while reducing side effects. Liposomal nanosystems and hydrogels have gained particular attention due to their versatility in encapsulating and regulating the release of active substances.

Liposomal nanosystems offer unique advantages for encapsulating bioactive compounds, protecting them from degradation, enhancing absorption, and providing targeted delivery. In the context of metabolic diseases, liposomal formulations have been explored for delivering insulin and insulin analogs [4,5], biguanide used in diabetes (e.g., metformin), lipid-lowering agents, including statins (e.g., atorvastatin, simvastatin [6,7] and fibrates (e.g., fenofibrate) [8], sulfonylureas (e.g., glibenclamide) [9], and anti-obesity drugs [10,11]. Additionally, several plant-derived bioactives have been successfully encapsulated in liposomal nanosystems, including resveratrol (grapes, berries), genistein (soy), guggulsterone (*Commiphora wightii*), berberine (*Rhizoma coptidis*), curcumin (turmeric), epigallocatechin gallate (green tea), and piperine (black pepper) for obesity management. Liposomal encapsulation improves solubility, stability, and bioavailability, offering a promising anti-obesity therapeutic platform [12]. Moreover, several therapeutic enzymes have been successfully encapsulated in liposomes to enhance their stability, circulation time, and efficacy in the prevention and treatment of metabolic and inflammatory diseases. These include L-asparaginase (originally used for leukemia, but also explored for metabolic regulation via amino acid depletion), catalase (antioxidant that degrades hydrogen peroxide), superoxide dismutase (used in management of metabolic syndrome, diabetic complications, and obesity-related inflammation), etc. [13]. Therefore, liposomal formulations offer a versatile platform to optimize pharmacokinetics, reduce toxicity, and improve therapeutic outcomes in metabolic disease management.

Hydrogels are also nutrient delivery systems that can increase the bioavailability, stability, and efficacy of bioactive compounds, potentially improving the prevention and treatment of metabolic and nutritional diseases. Hydrogels are regarded as an appealing biomaterial for a variety of medical applications due to their water absorption, soft structure, biocompatibility, and resemblance to extracellular matrix (ECM).

Hydrogels are functionalized for the controlled delivery of drugs, cells, or bioactive molecules. Thus, for application in metabolic diseases (e.g., diabetes, obesity, dyslipidemias, and metabolic liver diseases), they are being investigated as modern solutions for targeted and regenerative therapies [14].

Hydrogels show promise in treating metabolic diseases, particularly diabetes and metabolic syndrome, through various approaches, including glucose-responsive insulin delivery systems, which release insulin in response to high blood sugar. In the case of type 1 and 2 diabetes, injectable hydrogels with pancreatic β cells or islets of Langerhans can be used by protecting the transplanted cells from the immune response and releasing insulin depending on the glucose level [15,16].

Some hydrogels studied for the prevention and treatment of metabolic diseases can be mentioned, namely: (i) glucose-sensitive hydrogels, for example, poly (N-isopropylacrylamide) (PNIPAAm) combined with concanavalin A or phenylboronic acid; (ii) gastro-expandable hydrogels, for example, Plenity^®^ (Gelesis100)—FDA approved. Hydrogels containing chitosan or hyaluronate and growth factors (VEGF, EGF) promote diabetic wound healing. We can also mention hydrogels made from alginates or poly (vinyl alcohol) that are used to prolong the release of metformin, pioglitazone, or atorvastatin [17,18].

Adipose tissue dysfunction in obese people is associated with metabolic imbalances. To combat obesity, the development of a therapeutic hydrogel formed subcutaneously in situ (RL lip@gel) was investigated, which can modulate the inflammatory environment of adipose tissue and induce adipocyte browning, to combat obesity [19].

Both liposomal nanosystems and hydrogels provide significant benefits in the delivery of nutrients and bioactive compounds for the prevention and treatment of metabolic and nutritional diseases. The choice between the two systems depends on the nature of the compound, the route of administration, and the intended application (pharmaceutical vs. food). For example, Haishan Wu and his team [20] conducted a comparative study of oral insulin administration using liposomes and alginate hydrogels. Liposomes (Lip) containing arginine-insulin complexes (AINS) were incorporated into a hydrogel made from cysteine-modified alginate (Cys-Alg) to create liposome-in-alginate hydrogels (AINS-Lip-Gel). In vivo studies revealed that AINS-Lip-Gel released insulin in a controlled manner and had strong hypoglycemic effects. As a result, AINS-loaded liposomes in alginate hydrogels are a promising strategy for oral insulin delivery.

Liposomes are better suited for liposoluble compounds requiring efficient absorption, whereas hydrogels provide more versatile and cost-effective solutions for food and functional applications.

This paper discusses recent advances in the characterization, advantages, and limitations of liposomal nanosystems versus hydrogels as metabolic disease treatment systems.

## 2. Liposomal Nanosystems

Liposomal nanosystems encompass a diverse array of vesicular carriers tailored for efficient drug delivery in metabolic diseases, including liposomes, PEGylated stealth liposomes, stimuli-responsive liposomes and hybrid carriers combining liposomes with various nanomaterials.

Liposomes, also known as conventional or simple liposomes, are spherical structures composed of one or more natural, non-toxic phospholipid bilayers that enclose an aqueous compartment [21].

Liposome properties vary greatly depending on the lipid composition, surface charge, size, and preparation method [21].

They are widely used in drug delivery, vaccines, and gene delivery due to their biocompatibility and ability to protect active components.

### 2.1. Classification, Structure and Function of Liposomes

Liposomes are classified into three types based on size and number of bilayers: multilamellar, large unilamellar, and small unilamellar vesicles. Conventional liposomes, pH-sensitive liposomes, cationic liposomes, long-circulating liposomes (LCL), and immunoliposomes are all classified according to their composition. They are classified based on the preparation method as reverse-phase evaporation vesicles, French press vesicles, and ether injection vesicles [21].

Liposomes range in size from very small (0.025 μm) to large (2.5 μm). Furthermore, liposome membranes can be single or double-layered. The size of the vesicle is an important parameter in determining the circulating half-life of liposomes, and both the size and the number of bilayers influence the degree of drug encapsulation in liposomes [22].

Liposomes are classified into two types based on their size and number of bilayers: (1) multilamellar vesicles (MLV) and (2) unilamellar vesicles. Unilamellar vesicles are also divided into two types: large unilamellar vesicles (LUV) and small unilamellar vesicles (SUV) [23]. Unilamellar liposomes contain a single phospholipid bilayer sphere that surrounds the aqueous solution.

The vesicles in multilamellar liposomes resemble an onion. Typically, several smaller unilamellar vesicles are formed inside each other, forming a multilamellar structure of concentric phospholipid spheres separated by water layers [24].

The amphiphilic nature of liposomes allows simultaneous encapsulation of hydrophilic molecules (within the aqueous phase) and hydrophobic compounds (within the lipid bilayer). This dual capacity makes liposomes versatile carriers for a wide range of drugs. As a result, they act as transport systems, protecting sensitive drugs from enzymatic and chemical degradation while also allowing for controlled and targeted drug release in the body (for example, at the level of a tumor). They serve the purpose of reducing drug toxicity by encapsulating those with severe side effects (for example, doxorubicin) and preferentially targeting them to diseased tissues, reducing their effects on healthy tissues.

Liposomes can deliver DNA, RNA, and antigens into cells. Some mRNA vaccines (e.g., anti-COVID-19 vaccines) use lipid nanoparticles (similar to liposomes) to protect and deliver messenger RNA to cells [25].

Liposomal membranes mimic biological cell membranes, promoting biocompatibility and facilitating cellular uptake via fusion or endocytosis [26]. PEGylated stealth liposomes represent an advanced iteration of liposomal carriers, where polyethylene glycol (PEG) chains are covalently attached to the liposome surface to create a hydrophilic “stealth” coating that shields them from rapid clearance by the reticuloendothelial system, thereby extending circulation time in the bloodstream and improving systemic bioavailability [26].

**Figure 1:**
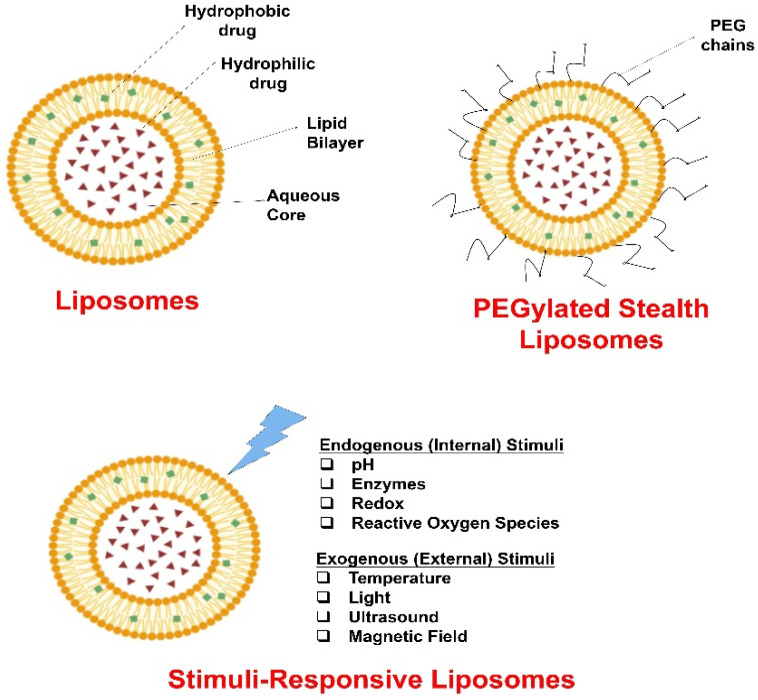
Liposomes vs. PEGylated Stealth Liposomes vs. Stimuli-responsive liposomes.

Stimuli-responsive liposomes (Figure 1) are engineered carriers that release their encapsulated payloads in response to specific environmental triggers, such as pH changes, temperature shifts, or enzymatic activity, allowing for precise, site-specific delivery that minimizes off-target effects and enhances therapeutic efficacy in nutritional interventions [27]. For instance, pH-sensitive liposomes can destabilize in the acidic milieu of the stomach or inflamed tissues to discharge drugs [28], while temperature-responsive liposomes might activate in febrile conditions associated with metabolic stress; this smart functionality boosting bioavailability [29].

Hybrid carriers combining liposomes with various nanomaterials, such as polymers, micelles, or nanoparticles, integrate the strengths of multiple delivery platforms to create multifunctional systems capable of dual or multi-modal encapsulation, overcoming limitations of single-component liposomes like instability or low loading capacity. These versatile constructs are liposome–polymer hybrids including liposome-in-hydrogel, liposome-in-film, and liposome-in-nanofiber, biopolymer-incorporated liposomes, guest-in-cyclodextrin-in-liposome, as well as liposome–inorganic nanoparticle hybrids. All can enable co-delivery of synergistic substances for the management of metabolic diseases. These systems enhance targeted absorption and controlled release, improving therapeutic outcomes by protecting bioactives from degradation and ensuring precise delivery to affected tissues [30,31].

### 2.2. Preparation of Liposomal Nanosystems

The preparation of liposomes and liposomal nanosystems is an important topic in pharmaceutical technology and medical nanotechnology.

**Figure 2:**
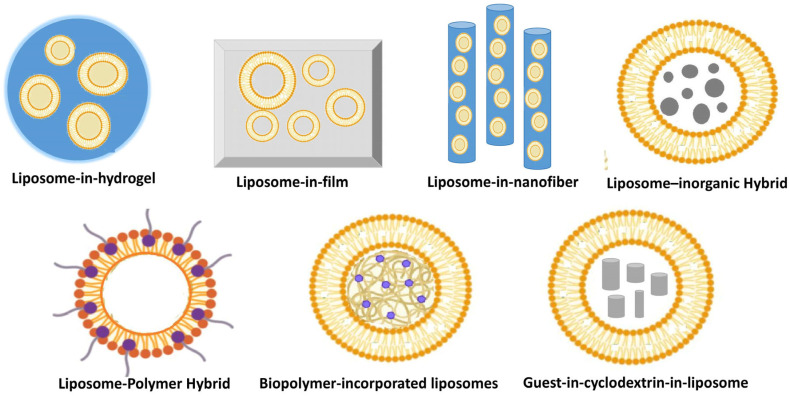
Liposomal Hybrid Nanosystems.

Liposomal nanosystems (Figure 1 and Figure 2) can be prepared through a variety of techniques; preparation technique directly dictates key attributes (size, lamellarity, polydispersity, encapsulation efficiency and stability), also the method selected must align with the intended application (e.g., intravenous formulation, topical nanocarrier, etc.).

Liposome preparation methods are generally classified into two broad categories: active loading techniques and passive loading techniques.

In active loading methods, liposomes are typically formed using approaches such as proliposome technology or lyophilization, where phospholipids are transferred from an organic phase into an aqueous phase. These methods involve the post-formation loading of drugs into preformed liposomes, often utilizing concentration gradients—such as pH or ammonium sulfate gradients—to drive the encapsulation of active compounds.

On the other hand, during the passive loading process, drugs are entrapped passively, therefore the drugs are incorporated during the liposome formation, not after.

The passive loading techniques include: (i) mechanical dispersion methods—these methods involve the dispersion of phospholipids in an aqueous medium via physical means and include: thin-film hydration (Bangham method), sonication, French press (extrusion); (ii) solvent dispersion methods such as: ethanol injection, ether injection (reverse-phase evaporation); and (iii) detergent removal methods these involve the utilization of surfactants to solubilize lipids and then their removal to allow vesicle formation. All of these methods are well described in the literature [21,32,33].

These preparation methods have been applied to obtain several liposomes for prevention and treatment of metabolic diseases, including obesity, diabetes, and dyslipidemia. For instance, glucagon-modified liposomes encapsulating triiodothyronine (T3) were prepared via thin-film hydration followed by extrusion, with glucagon modification for liver targeting. These liposomes (90–110 nm) exhibited high encapsulation efficiency (82.9%) and sustained T3 release, effectively improving metabolic outcomes in obese and hyperlipidemic mice [10].

Radziunas-Salinas et al. (2025) prepared dipalmitoyl phosphatidylcholine (DPPC) liposomes containing a long-chain cationic gemini surfactant, GS14, for atorvastatin delivery by solvent evaporation, hydration, and sonication [6]. The incorporation of GS14 enhanced drug loading and membrane stability, resulting in uniform unilamellar vesicles (~100 nm) [6]. Chen et al. (2009) reported soy phosphatidylcholine (SPC)-based liposomes prepared using the dry-film dispersion, sonication, and homogenization method, with either sodium deoxycholate (SPC/SDC) or cholesterol (SPC/CL) incorporated into the bilayer [8]. Systems had sizes of 120–200 nm and entrapment efficiency higher than 95% obtained at high SDC content attributed to the lipophilic affinity of fenofibrate and the solubilizing capacity of SDC within the bilayer [8].

Apart from these methods, supercritical fluid-assisted techniques offer green, efficient alternatives. These methods minimize solvent use, reduce toxicity, and are suitable for thermo-sensitive compounds. Supercritical CO_2_ is the most widely used fluid due to its non-toxic, non-flammable, and environmentally friendly nature, with mild critical conditions and low energy requirements. Liposomes produced via supercritical fluid-assisted techniques using CO_2_ methods exhibit enhanced uniformity, intactness, and controlled size compared to conventional methods. By adjusting parameters like decompression rate and nozzle diameter, liposome characteristics can be precisely tailored [34].

Literature on liposome prepared by supercritical fluid-assisted techniques for metabolic applications is notably scarce, with most studies focusing on general drug delivery or other therapeutic areas. A compelling exception is a PhD Thesis by Santo (2015) reporting the development of insulin-loaded nanometric liposomes using the SuperLip process, a continuous supercritical CO_2_-based method with high encapsulation rates (70–75%) and stable formulations tailored for oral administration in type 1 and type 2 diabetes, demonstrating the potential scalability and efficacy of this green technology for addressing metabolic challenges [35].

PEGylated “stealth” liposomes preparation involves PEGylation, the grafting polyethylene glycol chains onto the liposome surface, sterically hinders protein adsorption (opsonization) and reduces RES uptake, thereby prolonging systemic circulation. PEGylated lipids (e.g., DSPE-PEG2000) can be introduced by co-formulation. In co-formulation the PEGylated lipids are included in initial lipid mixture before film formation or solvent injection, producing uniform distribution in the membrane. This is the most common route for clinical formulations.

Another method to obtain PEGylated “stealth” liposomes is post-insertion (micellar transfer), where the pre-formed PEGylated-lipid micelles are incubated with preformed liposomes; PEG-lipids insert into the bilayer driven by hydrophobic interactions. This can be used to modify surface density after liposome formation or to add PEG with different terminal chemistries for ligand conjugation. PEGylation also impacts membrane packing and drug release kinetics, so optimization is formulation-specific [36].

Maritim et al. (2021) reported PEGylated glibenclamide liposomes produced via ethanol injection and calcium acetate remote loading, with PEGylation and lipid optimization [9]. Particle size (~130 nm) and encapsulation efficiency were tunable via lipid chain length, saturation, cholesterol content, and drug-to-lipid ratio, achieving controlled release, high stability, and optimized glibenclamide delivery for diabetes management [9]. Also, lyophilized long-circulating PEGylated liposomes with simvastatin were developed through PEGylation, extrusion, and freeze-drying under a Quality by Design framework, for parenteral use in metabolic diseases treatment. Three formulation factors, like PEG proportion (0–5%), cholesterol concentration (5–15 mM), and the cryoprotectant-to-phospholipid molar ratio (0–5) and two process parameters like number of extrusions (1–3 passes) and lyophilization conditions (−80 °C, −20 °C, or none) were investigated to assess their effect on liposomal size, encapsulated simvastatin concentration and retention of drug. The study revealed that cholesterol concentration had the most pronounced effect among the formulation factors, critically influencing vesicle stability, membrane rigidity, and drug retention. PEG proportion also contributed to liposome stability and long-circulating properties. The particle size of these nanosystems was consistently maintained within the target range (~100–150 nm), with high encapsulation efficiency and excellent drug retention. This approach demonstrated that QbD enables a rational, time-efficient, and scientifically robust strategy for developing lyophilized liposomal formulations with predictable and controlled quality, offering enhanced stability, reproducibility, and suitability for parenteral administration of simvastatin [7].

Stimuli-responsive liposomes are engineered to trigger release in response to internal signals (pH, redox, and enzymes) or external stimuli (temperature, light, magnetic fields, and ultrasound). Preparation generally follows conventional vesicle formation with incorporation of specialized lipids or functional moieties [27].

In the case of liposome–polymer hybrids preparation approaches include encapsulation of polymeric cores inside lipid shells, surface coating of liposomes with polymers (e.g., chitosan, hyaluronate, alginate) or layer-by-layer polyelectrolyte assembly [30]. For example, modified-release insulin-loaded liposomes coated with poloxamer F188 and PEG500 were fabricated via the heating method with particle sizes of 76.6–155.0 nm and encapsulation efficiencies of 17.61–76.08% administered via subcutaneous (SC) or oral (PO) routes in streptozotocin-induced type 1 diabetic Wistar rats [4]. Similarly, Song et al. (2025) developed a novel bilayer liposome system for oral insulin delivery, consisting of an inner soybean phosphatidylcholine-cholesterol liposome (4:1 *w*/*w*) encapsulating insulin, coated with propanoic acid-functionalized chitosan (CS/PA) [5]. The liposomes were prepared via thin-film hydration followed by probe sonication, with CS/PA decoration achieved by dropwise addition of the insulin-loaded liposomes into a 0.5% (*w*/*v*) CS/PA solution under gentle stirring. This bilayer system significantly improved gastrointestinal stability and targeted MCT1-mediated intestinal absorption, demonstrating superior hypoglycemic efficacy over uncoated liposomes [5].

In the case of liposome–inorganic nanoparticle hybrids inorganic nanoparticles (gold, iron oxide, silica, quantum dots) can be (i) encapsulated in the aqueous core, (ii) embedded within the bilayer (hydrophobic coated NPs), or (iii) surface-attached via linkers.

Preparation is application-specific and major challenges include: ensuring reproducible scale-up and regulatory compliance, preventing accelerated clearance due to anti-PEG immune responses, controlling long-term stability (aggregation, leakage), and thoroughly characterizing hybrid materials’ toxicology [37].

### 2.3. Stability and Bioavailability of Liposomal Nanosystems

The stability of liposomes are not inherent properties of the carrier structure alone, but result from the strategic manipulation of formulation composition and preparation methods [7,9,38]. Optimizing factors like lipid type, cholesterol concentration, surface charge, and drug location is critical for achieving desired physicochemical properties, including size, polydispersity index (PDI), zeta potential (ZP), encapsulation efficiency (EE%), and release kinetics ultimately determining in vivo performance [7,9].

#### 2.3.1. The Critical Role of Cholesterol and Lipid Composition

The stability of liposomes is influenced by lipid composition, storage conditions, pH, and the presence of cholesterol, which modulates membrane rigidity. Encapsulation protects sensitive bioactives from oxidation, hydrolysis, or enzymatic degradation [38]. Liposomal carriers improve bioavailability by facilitating solubilization of poorly water-soluble molecules, enhancing intestinal absorption, and bypassing first-pass metabolism [39]. Cholesterol is a highly influential component commonly added to liposome formulations to promote membrane rigidity, drug loading, and colloidal stability [9,40]. The impact of cholesterol concentration on liposome properties varies significantly based on its effect on the membrane structure and the location of the encapsulated drug [9]. Increasing cholesterol content generally improved particle uniformity (lowered PDI), imparting greater stability by intercalating between lipid chains and promoting structural packing. However, increased cholesterol content can contribute to an undesirable increase in liposomal size [7]. In studies involving neutral lipids (DPPC), neither cholesterol nor the drug had a significant impact on the ZP in the concentration range investigated [9]. For drugs incorporated directly into the lipid bilayer (e.g., glibenclamide or simvastatin), increasing cholesterol content generally resulted in a decreased EE% and drug loading (D/L) [7,9]. For weakly acidic drugs encapsulated in the aqueous core using remote loading (e.g., glibenclamide), increasing the cholesterol amount increased the final drug loading. For example, a formulation at a 2:1 PC:cholesterol ratio achieved an 8-fold higher loading compared to a 5:1 PC:cholesterol ratio, because cholesterol increases the hydrophobicity of the bilayer, reducing permeability and enhancing retention of the ionized drug in the core [9]. Increasing the amount of cholesterol generally accelerated the rate of drug release for formulations where the drug was incorporated in the bilayer (e.g., HSPC liposomes).

Lipid saturation and acyl chain length are critical determinants of the lipid phase transition temperature (*Tc*), which profoundly influences membrane rigidity, EE%, stability, and release kinetics [9,41]. Higher EE% and drug loading were consistently achieved using lipids with longer acyl chains (e.g., 90.3% EE for HSPC). For saturated lipids, EE% increased with chain length, ranking as DMPC (14C) < 15:0 PC (15C) < DPPC (16C) < HSPC (18C). Liposomes made from lipids with longer chains (e.g., DSPC, 18C) exhibited a decreased rate of drug release and prolonged the release duration [9]. This effect is due to the higher *Tc* of these lipids (DSPC *Tm* ≈ 41 °C) [7], ensuring the lipid bilayer exists in a rigid gel state above the physiological temperature (T > 37 °C), thereby maintaining stability and superior drug retention. Conversely, introducing a higher degree of lipid unsaturation dramatically reduces *Tc* [9,42]. Liposomes made from unsaturated lipids (low *Tc*, e.g., DOPC at −15 °C) exist in a fluid state at physiological temperature, resulting in a faster drug release rate and lower retention.

#### 2.3.2. The Impact of Surface Modification and Ligands

Surface modifications, such as PEGylation or ligand conjugation, further extend circulation time and enable tissue-specific targeting [5,6,10,43]

*PEGylation*. Although liposomes have been widely studied for the delivery of drugs and nutraceuticals in metabolic diseases, their colloidal stability and in vivo bioavailability remain limiting factors [44]. Conventional liposomes are prone to aggregation, fusion, oxidation of phospholipids, and hydrolysis, leading to reduced shelf life and unpredictable pharmacokinetics [45]. In the gastrointestinal tract, liposomes encounter bile salts, digestive enzymes, and variable pH, which compromise their structural integrity and thus reduce the oral bioavailability of encapsulated bioactives such as insulin, curcumin, and other peptides [46].

PEGylation, the covalent grafting of polyethylene glycol (PEG) chains onto the liposomal surface, is one of the most established strategies to increase stability and circulation time [47]. PEG forms a hydration shell that reduces opsonization by serum proteins and recognition by the reticuloendothelial system (RES), thereby prolonging the plasma half-life of liposomes [47]. PEGylated liposomes have demonstrated improved delivery of substances, showing enhanced pharmacokinetics and reduced off-target toxicity in disease models [36]. However, PEGylation can trigger “accelerated blood clearance” (ABC) phenomena upon repeated dosing and may hinder cellular uptake, which motivates further optimization with cleavable PEG linkers or mixed-surface modifications [48,49].

*Surfactants*. The incorporation of long-chain cationic gemini surfactants (14-14-14) into dipalmitoyl phosphatidylcholine (DPPC) liposomes was found to be highly effective in boosting stability and drug retention for anionic drugs like atorvastatin [6]. The surfactant inclusion drastically reduced the average hydrodynamic size (e.g., from ~900 nm to ~80 nm) and the PDI, suggesting a more uniform structure. Furthermore, the cationic nature conferred great electrostatic stability by significantly increasing the Zeta Potential (ZP) (e.g., from 5.7 ± 0.2 mV to 49.1 ± 1.8 mV). This approach also nearly doubled the retention percentage of atorvastatin compared to conventional liposomes, driven by polar interactions between the cationic surfactant and the anionic drug [6]. Differential Scanning Calorimetry (DSC) confirmed that the gemini surfactants altered the stability, causing a slight decrease and broadening of the phase transition temperature (*Tm*), indicating disruption of the bilayer organization [6]. Another example reported the inclusion of bile salts, such as Sodium Deoxycholate (SDC), in fenofibrate liposomes creating specialized transfersomes [8]. SDC incorporation was shown to increase membrane fluidity and was directly implicated in the enhanced oral bioavailability, resulting in a 5.13-fold higher bioavailability compared to micronized capsules [8]. The mechanism relies on the ability of the bile salt-containing liposomes to readily transform into mixed micelles in the gastrointestinal environment, facilitating enhanced transmembrane absorption, despite exhibiting a slower in vitro release profile than the micronized.

*Targeting Ligands for Specific Tissues*. Targeting ligands represent powerful tools used to enhance nanocarrier specificity and stability in complex in vivo environments, a strategy crucial for delivering fragile therapeutic agents and minimizing systemic toxicity [10,11]. For instance, in anti-obesity therapy, researchers focused on improving the stability and specificity of the novel peptide PDBSN (GLSVADLAESIMKNL), whose intended use was hampered by poor stability during blood circulation [11]. The solution involved encapsulating PDBSN into liposomes that were subsequently attached to two crucial ligands: a cell-penetrating peptide (CPP) and a visceral-adipose-tissue-targeting peptide (CGLHPAFQC). This targeted formulation was successfully prepared with high encapsulation efficiencies for the constituent parts (PDBSN: 63.14%; targeting peptide: 69.71%) [11]. The targeting modification proved highly successful in vivo, resulting in the robust enrichment of the peptide in adipose tissue, particularly visceral fat, where the fluorescence intensity was observed to be almost 5-fold higher than in other adipose tissues, confirming the efficacy of the CGLHPAFQC ligand [11]. This enhanced stability and specificity allowed the liposome-encapsulated PDBSN to successfully reduce weight gain and improve metabolic homeostasis in obese mouse models. Similarly, glucagon modification was employed to achieve precise organ targeting for the potent anti-obesity agent, Thyroid Hormone (T3) [10]. T3 effectively influences energy expenditure and fat oxidation, but its broad distribution of thyroid hormone receptors can lead to severe side effects, notably cardiovascular and bone thyrotoxicity. To minimize these systemic risks, Glucagon-modified liposomes (GCG-lipo/T3) were developed to deliver T3 selectively to key metabolic organs, specifically the liver and adipose tissues. This modification was pivotal for enhancing stability and targeting. Characterization of the GCG-lipo/T3 formulation showed that the glucagon modification led to a significantly higher Encapsulation Efficiency (EE %) of 82.9 ± 5.3% compared to the unmodified T3-loaded liposome (63.0 ± 4.1%) [10]. Crucially, this improvement in loading capacity was accompanied by a desirable reduction in the leakage rate (4.9 ± 0.3% versus 13.3 ± 0.8%). In vivo biodistribution imaging confirmed that the GCG-lipo was efficiently enriched in the liver, demonstrating that the targeted delivery strategy successfully minimized unwanted exposure to the cardiovascular system and bone [10].

These modifications extend circulation time, enable tissue-specific targeting, and mitigate aggregation, fusion, and phospholipid oxidation—common failure modes of conventional liposomes [44,45].

#### 2.3.3. Stimuli-Responsive and Hybrid Designs

Stimuli-responsive liposomes have been engineered to overcome premature release and poor site-specific delivery, pH-sensitive liposomes are particularly relevant for diseases involving microenvironments mildly acidic [50].

Redox-responsive liposomes exploit the high intracellular glutathione levels in hepatocytes or adipocytes to achieve controlled cytosolic release of bioactives [51].

Enzyme-responsive liposomes (e.g., lipases, matrix metalloproteinases) provide selective drug release in tissues undergoing remodeling, as seen in obesity-induced adipose inflammation and Non-Alcoholic Fatty Liver Disease (NAFLD) fibrosis [52]. These adaptive designs improve target-site bioavailability, reduce systemic exposure, and enhance therapeutic efficacy in preclinical models [27,50].

Liposomal hybrid materials exhibit improved structural stability, sustained release, and enhanced oral absorption of poorly soluble drugs and nutraceuticals relevant to obesity and NAFLD.

Hybrid systems like polymer coating liposomes provide mucoadhesive surfaces, pH-responsive shielding, and transporter-targeted uptake, collectively overcoming gastrointestinal barriers to achieve markedly higher oral bioavailability [4,5]. For insulin delivery, liposomes coated with propanoic acid-functionalized chitosan (CS/PA) were synthesized to target the Monocarboxylate Transporter 1 (MCT1) in the intestine [5]. The CS/PA decoration successfully shifted the liposome’s ZP to a highly negative value (−25.31 ± 6.60 mV), enhancing stability. Furthermore, the coating provided superior protection for insulin in simulated gastric fluid (SGF) and was confirmed to increase intestinal permeability and absorption by mediating uptake via MCT1-mediated energy-dependent endocytosis. This strategy ultimately yielded a relative oral bioavailability of 9.82 ± 0.56% compared to subcutaneous injection [5]. Also, Chávez-Corona et al. (2025) fabricated modified-release insulin-loaded liposomes coated with poloxamer F188 and PEG500 administered via subcutaneous or oral routes in streptozotocin-induced type 1 diabetic Wistar rats [4]. By the subcutaneous route, formulation demonstrated modified-release profiles, inducing hypoglycemia earlier (35–240 min) than commercial insulin (Insulex^®^, USA; 60–120 min), and exhibiting pronounced and sustained glucose reduction. Oral administration proved ineffective, with glucose remaining >300 mg/dL across prototypes, prompting recommendations for future incorporation of chitosan to enhance mucoadhesion, gastrointestinal stability, and oral bioavailability. No toxicity was observed, with normal liver and kidney function analytes, body weight maintenance, and unremarkable histological findings over 15 days [4].

Liposome–in-hydrogel composites made by combining liposomes with hydrogels is a strategy used to harness the benefits of both delivery systems, particularly for oral applications [31,53]. Encapsulating insulin-loaded liposomes within an alginate hydrogel matrix was shown to promote the oral bioavailability of insulin. The hydrogel packaging is essential, as it functions to suppress the initial burst release of the drug and protects the liposomes’ structural integrity from degradation in the acidic environment of the gastrointestinal tract [53].

Additionally, liposome–metal hybrids allow for external control of drug release (magnetic or photothermal stimuli), which has been explored for targeted therapies. These systems hold promise for multimodal therapies, where therapeutic delivery can be combined with external control for precision treatment of complex disorders [30].

### 2.4. Advantages and Limitations of Liposomal Nanosystems

Liposomal nanosystems offer a versatile platform for the prevention and treatment of metabolic diseases, with each type presenting unique advantages and limitations. In Table 1 are presented the advantages and limitations of liposomal nanosystems relevant for metabolic diseases prevention and treatment.

Conventional liposomes, composed of phospholipid bilayers, are biocompatible, can encapsulate both hydrophilic and lipophilic drugs, and protect therapeutics from enzymatic degradation, making them suitable for delivering insulin, curcumin, silibinin, or lycopene in diabetes, obesity, and NAFLD. However, they are prone to oxidation, hydrolysis, fusion, and rapid clearance by the reticuloendothelial system, limiting their oral bioavailability [54,55,56].

PEGylated “stealth” liposomes, modified with polyethylene glycol, improve colloidal stability, prolong circulation time, reduce opsonization, and enhance pharmacokinetics for drugs such as statins, siRNA, and antioxidants in metabolic syndrome, dyslipidemia, and liver diseases. Their limitations include the accelerated blood clearance effect upon repeated dosing, potential hindrance of cellular uptake, and immunogenicity concerns [57,58,59].

Stimuli-responsive liposomes, engineered to release payloads in response to pH, redox changes, enzymes, or external triggers, provide controlled, site-specific drug delivery, reducing systemic toxicity and improving local bioavailability. Nonetheless, these systems are complex to manufacture, may show variable in vivo trigger efficiency, and can exhibit lower storage stability [60,61].

Finally, hybrid liposomal carriers, incorporating polymers, metals, or silica, offer enhanced structural stability, higher drug-loading efficiency, sustained release, and potential for multimodal therapy, including liver-targeted or oral delivery of hydrophobic nutraceuticals. These advantages are counterbalanced by complex, costly synthesis, regulatory hurdles, and uncertain long-term safety [62,63].

Collectively, these nanosystems demonstrate significant potential for improving therapeutic outcomes in metabolic diseases, although careful consideration of their limitations is critical for clinical translation.

### 2.5. Applicability of Liposomal Nanosystems in the Prevention of Metabolic Diseases

**Figure 3:**
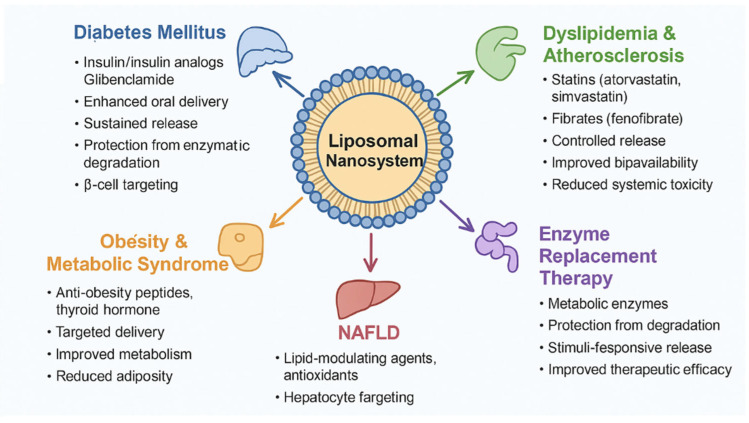
Applicability of liposomal nanosystems.

Liposomal nanosystems provide a multifaceted platform for the prevention and treatment of metabolic diseases. By improving pharmacokinetics, enhancing tissue targeting, reducing toxicity, and allowing for controlled release, liposomal nanosystems have the potential to revolutionize therapy for diabetes, dyslipidemia, obesity, NAFLD, and enzyme deficiency disorders, etc. (Figure 3).

#### 2.5.1. Diabetes Mellitus

Diabetes mellitus encompasses a group of metabolic disorders characterized by chronic hyperglycemia due to defects in insulin secretion, insulin action, or both. In type 1 diabetes mellitus (T1DM), an autoimmune-mediated destruction of pancreatic β-cells leads to absolute insulin deficiency. In type 2 diabetes mellitus (T2DM), insulin resistance and relative insulin deficiency predominate. Both conditions necessitate effective glycemic control to prevent complications such as neuropathy, nephropathy, retinopathy, and cardiovascular diseases.

Liposomal formulations have been extensively explored for the delivery of antihyperglycemic agents, offering advantages in terms of bioavailability, stability, and controlled release. Modified-release insulin-loaded liposomes have demonstrated effective glycemic control in experimental T1DM models. These formulations provide sustained insulin release, reducing the frequency of administration and improving patient compliance. Additionally, glucose-responsive multivesicular liposomes (MVLs) have been developed, which release insulin in response to elevated blood glucose levels, mimicking physiological insulin secretion [64].

Glibenclamide-loaded liposomes have been investigated for their stability and sustained release, potentially improving efficacy and minimizing gastrointestinal side effects. Research indicates that liposomal formulations can enhance the oral bioavailability of glibenclamide, offering a non-invasive alternative to traditional therapies [65].

In summary, liposomal nanosystems represent a promising approach in the prevention and treatment of diabetes mellitus, offering enhanced delivery of antihyperglycemic agents, improved patient compliance, and potential for personalized therapy.

#### 2.5.2. Dyslipidemia and Atherosclerosis

Hyperlipidemia, characterized by elevated levels of lipids in the blood, is a significant contributor to the development of atherosclerosis and cardiovascular diseases [66]. Liposomal formulations of lipid-lowering agents, such as statins and fibrates, have been extensively studied to enhance their therapeutic efficacy and reduce systemic side effects.

Statins, including atorvastatin and simvastatin, are commonly prescribed to manage hyperlipidemia by inhibiting HMG-CoA reductase, the rate-limiting enzyme in cholesterol biosynthesis. However, their clinical efficacy can be limited by poor solubility and rapid systemic clearance [6,7,67].

Fibrates, such as fenofibrate, are utilized to reduce triglyceride levels and increase high-density lipoprotein (HDL) cholesterol. Fenofibrate’s poor water solubility poses challenges for its oral bioavailability. Liposomes incorporating bile salts, such as sodium deoxycholate, have been shown to significantly enhance the solubility and bioavailability of fenofibrate. These formulations address the drug’s poor water solubility, facilitating improved absorption and therapeutic efficacy [8,68]. Therefore, liposomal nanosystems represent a promising approach in the prevention and treatment of dyslipidemia and atherosclerosis. By optimizing the delivery of lipid-lowering agents, these formulations can improve therapeutic outcomes and patient quality of life.

#### 2.5.3. Obesity and Metabolic Syndrome

Obesity is a major risk factor for T2DM, cardiovascular disease, and metabolic syndrome. Liposomal formulations offer an efficient strategy for delivering anti-obesity drugs and peptides. For example, glucagon-modified liposomes loaded with thyroid hormone have been shown to enhance fat metabolism and limit weight gain in preclinical models [51]. Similarly, liposome-encapsulated peptide PDBSN alleviates high-fat diet-induced obesity and restores metabolic balance by regulating lipid and glucose metabolism [57]. Beyond efficacy, liposomal encapsulation improves the stability of peptide-based drugs, protects them from rapid degradation, and enables targeted tissue delivery—highlighting its potential as a precision therapeutic approach for obesity [10,11,69].

#### 2.5.4. Non-Alcoholic Fatty Liver Disease (NAFLD)

NAFLD, encompassing simple steatosis to non-alcoholic steatohepatitis, is a metabolic disorder characterized by excessive fat accumulation in the liver, often linked to insulin resistance, obesity, and type 2 diabetes. Liposomal nanosystems are emerging as promising tools for NAFLD treatment due to their ability to encapsulate bioactive compounds, protect them from degradation, enhance bioavailability, and provide targeted delivery to hepatocytes or other liver cells. Liposomal nanosystems delivering lipid-modulating agents and antioxidants, such as statins, silybin, or resveratrol, target hepatocytes to effectively treat NAFLD by reducing oxidative stress, inflammation, and hepatic lipid accumulation [6,70,71]. These lipid-based liposomes enhance bioavailability and enable controlled release, improving hepatic lipid metabolism through pathways like AMPK and PPARγ, thus mitigating steatosis and fibrosis while offering a promising therapeutic strategy for NAFLD management [60].

#### 2.5.5. Enzyme Replacement Therapy and Metabolic Enzyme Delivery

Enzyme replacement therapy has become an important approach for treating metabolic disorders caused by enzyme deficiencies or dysregulation; however, its success is often hindered by challenges such as short plasma half-life, rapid enzymatic degradation, immunogenicity, and limited delivery to target tissues. Liposomal nanosystems provide unique advantages for encapsulating and delivering therapeutic enzymes by protecting them from inactivation, prolonging circulation time, and enhancing their uptake into specific tissues [49,72]. Several metabolic and antioxidant enzymes have been successfully encapsulated in liposomes, including L-asparaginase, which depletes circulating amino acids and has been studied not only for leukemia but also for metabolic regulation, as well as catalase and superoxide dismutase, which play critical roles in reducing oxidative stress, inflammation, and metabolic dysfunction linked to diabetes, obesity, and metabolic syndrome. By stabilizing these enzymes and facilitating controlled release, liposomal carriers enhance therapeutic efficacy and reduce systemic side effects, providing an attractive delivery strategy for enzyme-based therapies in metabolic and inflammatory diseases [13] (Table 2).

## 3. Hydrogels

Hydrogels have recently gained popularity in the prevention and treatment of metabolic diseases (such as diabetes, obesity, dyslipidemias, and metabolic syndrome) due to their role in controlled drug delivery, tissue regeneration, and biomarker monitoring.

### 3.1. Classification, Structure and Function of Hydrogels

**Figure 4:**
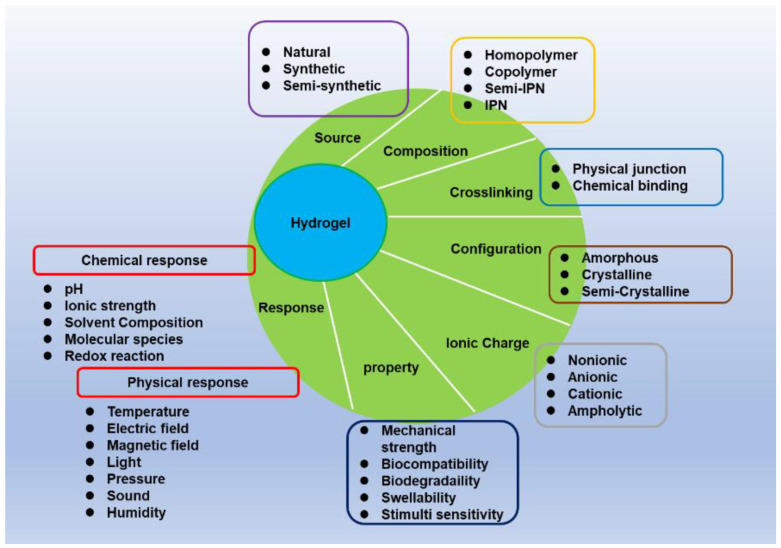
Classification of hydrogels [73].

Hydrogels can be classified based on their source, composition, environmental stimuli, crosslinking, properties, configuration, and ionic charge, as briefly illustrated in Figure 4.

Hydrogels can be made from natural, synthetic, or semi-synthetic polymers. Hydrogels can be classified according to their polymer source as natural, synthetic, or semi-polymeric. Natural hydrogels include cellulose, chitosan, collagen, alginate, agarose, hyaluronic acid, gelatin, and fibrin. They have inherent biocompatibility, bioactivity, and biodegradability, but their mechanical stability and strength are relatively low. Although natural hydrogels are safe for most people, some of them are allergenic in rare cases [73].

Synthetic hydrogels are constructed from synthetic polymers prepared by polymerization of a monomer, such as polyvinyl alcohol (PVA), polyethylene glycol (PEG), polyethylene oxide (PEO), poly-2-hydroxyethyl methacrylate (PHEMA), poly-N-isopropyl acrylamide (PNIPAM), polyacrylic acid (PAA), and polyacrylamide [74,75,76].

Semisynthetic polymers are chemically modified natural polymers or a blend of natural and synthetic polymers used to create semi-hydrogels. Examples of chemically modified natural polymers include methacryloyl-modified gelatin (GelMA) [77] and acrylate-modified hyaluronic acid (AcHyA) [78].

Hydrogels can respond physically, chemically, or biomedically. Physical, chemical, or biomedical stimuli have been shown to change the physical properties of hydrogels, such as deformation (the transition from swollen to shrunken hydrogel) and self-assembly [79,80,81,82,83]. These stimuli can be found in solvents or external environments.

The physical stimuli for hydrogels are temperature, pressure, light, electric fields, and magnetic fields [80,81], while the chemical stimuli are pH, ionic strength, solvent composition, and molecular species [80,82,83]. In addition, hydrogels exhibit biomedical stimuli such as antigenic responses, ligands, and enzymes [83].

Hydrogels are classified not only by their physical properties, but also as conventional hydrogels or smart hydrogels.

Conventional hydrogels change only slightly in response to external environmental conditions, resulting in swelling and low mechanical strength, whereas smart hydrogels are sensitive to small changes in external environmental conditions and immediately adjust their physical properties (such as mechanical strength, swelling, and sensitivity to stimuli) [79,80,81,82,83].

Research on hydrogel systems with structural features has aroused great interest due to their wide range of applications, including actuators, microfluidic units, synthetic adhesives, transplantable tissue organs, and cell scaffolds.

Hydrogels are soft materials composed of three-dimensional, insoluble, cross-linked polymer networks that can absorb a significant amount of water [84]. These polymer networks are frequently linked together through chemical or physical interactions.

Typical chemically cross-linked hydrogels are frequently produced through free radical polymerization initiated by heat, light, or radiation. In contrast, physically cross-linked hydrogels are formed by non-covalent bonding interactions at the molecular level.

Polymer hydrogel systems have found important applications in both everyday life and advanced industries [85]. Multi-networks, such as the double network hydrogel, are commonly used to create tough hydrogels due to the poor mechanical properties of single chemical cross-linked networks [86,87]. Research suggests that combining chemical and physical networks can create high-strength hydrogels [88].

**Figure 5:**
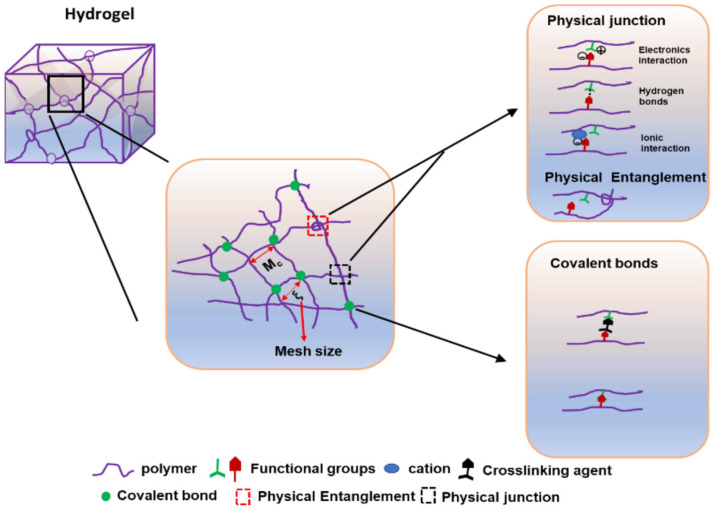
The structure of hydrogels (chemical linking and physical junctions) [73].

As illustrated in Figure 5, a covalent bond can be formed between two functional groups of polymers with or without covalent agents (via enzymatic catalysis). Electronic interactions between opposite charges in the polymer can cause the physical junction to form. It can also form hydrogen bonds or interact with ions in solution [89,90].

The hydrogel mesh retains water and has an elastic force, which is caused by swelling and water release [91]. As a result, the meshes can keep the hydrogels solid [91]. They play an important role in fluid exchange within the polymer network, as well as cell or drug transport [92]. The size of the mesh holes is known as mesh size, and it is related to the drug release time [81] and the linear distance between two crosslinking points [93,94].

Demeter M. et al. used rheological analysis to show how the average molecular weight between crosslinking points (Mc) and crosslinking density (Ve) affect mesh size. Mc is positively correlated with mesh size [95]. Steinman NY et al. found that 4 kDa and 8 kDa PEG can form 11 kDa and 16 kDa hydrogels at the same crosslinking agent concentration [96]. Thus, the molecular weight of hydrogels is practically determined by the polymer content, which can be infinite due to their 3D network structure [96,97].

The chemical structure and morphology of hydrogels influence properties such as mechanical strength and intracellular and extracellular transport [98].

**Figure 6:**
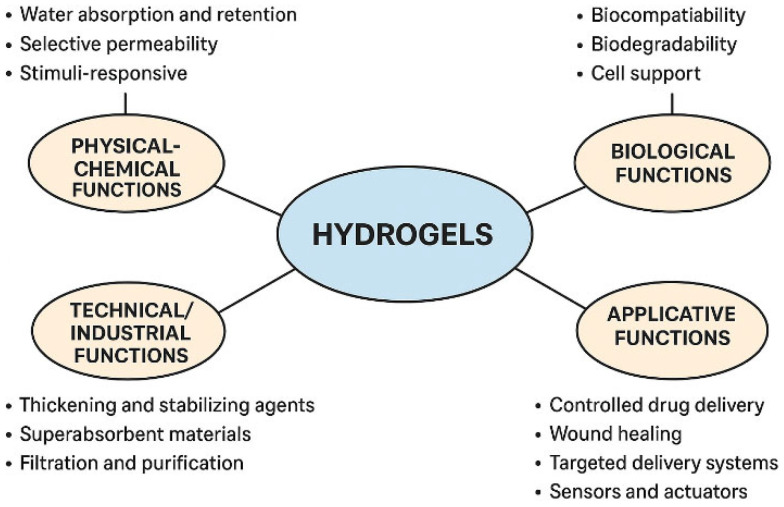
The main functions of hydrogels.

Hydrogels are particularly appealing for various medical applications due to their water absorption, soft structure, biocompatibility, reduced protein adsorption, low surface tension, and structural similarity to the ECM. These applications [99,100,101,102,103] include tissue engineering, therapeutic agent delivery (such as proteins, drugs, and genes), contact lenses, and wound dressings (see Figure 6).

Hydrogels can also be classified based on their intended applications, as shown in the examples in Table 3.

### 3.2. Preparation of Hydrogels

Hydrogels are three-dimensional materials formed from hydrophilic polymers that can absorb and retain large amounts of water while remaining structurally intact. They can be obtained from natural polymers (gelatin, alginate, and chitosan) or synthetic polymers (poly (ethylene glycol), poly (acrylamide), and poly (vinyl alcohol)) and are used in biomedical and pharmaceutical applications [104].

The preparation methods are classified based on the types of interactions that form the network (Figure 4 and Figure 5).

In the case of physical methods (non-covalent), the hydrogel is formed through weak interactions (hydrogen, ionic, or hydrophobic forces). For example, thermoreversible gelation (e.g., gelatin, poloxamers) and ionotropic gelation (e.g., sodium alginate with Ca^2+^ ion).

While chemical methods (covalent crosslinking) involve chemical reactions between the functional groups of polymers, using crosslinking agents (e.g., glutaraldehyde, EDC/NHS).

Other variants include radical polymerization or photo-induced crosslinking (photo-crosslinking), frequently used for injectable systems or stimuli-responsive gels.

Recently, researchers have focused more on hydrogels that respond to biological conditions [105]. Using this type of hydrogel, also known as injectable hydrogen, the patient is spared the complications of implant surgery, including pain and inflammation.

Natural hydrogels exhibit appreciable biocompatibility, but they have poor mechanical properties and lower stability [106].

Yang et al. used Schiff base crosslinking reactions to create an in situ hydrogel based on chitosan and hyaluronic acid [107]. Studies demonstrated that the hydrogels improved cellular responses and could be used as a permanent biomaterial for abdominal tissue engineering [108].

Synthetic hydrogels are more widely used than natural hydrogels due to their ease of fabrication and tailoring. The use of synthetic hydrogels can trigger changes in physicochemical as well as mechanical properties. They may have disadvantages such as biocompatibility issues with a limited number of polymers [109].

Hybrid hydrogels are hydrogels composed of both natural and artificial polymers. Several natural biopolymers have been combined with synthetic polymers to maximize the benefits of both natural and synthetic hydrogels [110]. Bonnet et al. presented the existence of an injectable PIVIPAAM, physically cross-linked polyethylene glycol hydrogel, which demonstrated sensorimotor function. The authors concluded that the fabricated hydrogels may be found to have suitable functions in axon regeneration.

### 3.3. Stability and Bioavailability of Hydrogels

Hydrogels are widely used in biomedical applications, including drug delivery, tissue engineering, and smart dressings. Their clinical and pharmaceutical applications require two critical properties: stability and bioavailability [99,100,101,102,103].

The stability of hydrogels is critical for applications in medicine, pharmacy, tissue engineering, agriculture, and the food industry. It describes the hydrogel’s ability to maintain its structural, physicochemical, and functional properties over time and under various operating conditions.

Several factors influence stability, including chemical composition, degree of crosslinking, mechanical properties, and degradability. Thus, crosslinking (physical or chemical) strengthens the polymer network and reduces dissolution in water. Chemical crosslinking is generally more stable than physical crosslinking.

pH and ionic strength influence the hydrogel’s swelling and integrity, while temperature can cause sol-gel transitions or degradation.

Hydrogels formed from natural polymers can be degraded by enzymes found in the biological environment.

Stability is also determined by compressive, shear, and tensile strength, all of which are important in biomedical applications.

Hydrogels can be biodegradable (for controlled drug release) or non-reactive (long-term stability).

Mechanical stability is commonly used to assess hydrogel strength by resisting external forces while maintaining structural integrity. Chemical stability is also determined by testing hydrogels’ resistance to specific hydrolysis, oxidation, and enzymatic reactions.

Studies have shown that natural hydrogels formed from polymers such as alginate, gelatin, collagen, or chitosan are more biocompatible but mechanically and chemically unstable, whereas synthetic hydrogels made from polyacrylamide, PEG, or PVA are more stable but may have limited biocompatibility [105].

The bioavailability of hydrogels refers to the amount and rate at which the active substance is released and available at the site of biological action.

Factors influencing bioavailability include: (i) diffusion of the drug through the hydrogel network, which is dependent on porosity, degree of hydration, and molecular interactions; (ii) controlled degradability of hydrogels that erode or degrade enzymatically, gradually releasing the substance; (iii) sensitivity to stimuli (pH, temperature, enzymes, and magnetic fields) allows targeted drug release; (iv) biocompatibility and non-toxicity can avoid side effects that reduce absorption; (v) local vs. systemic administration of injectable or implantable hydrogels can increase local drug concentration, thus improving bioavailability [111,112,113].

Drug administration in hydrogels has numerous advantages over other systems, including: (i) protecting the drug from premature degradation (for example, enzymatic degradation or gastric acid); (ii) allowing sustained release, extending the time of maintaining the therapeutic concentration; and (iii) reducing adverse effects through localized release.

Finally, the bioavailability of hydrogels is largely determined by the polymer composition, active substance type, and route of administration. In general, hydrogels have been shown to increase the bioavailability of drugs that are difficult to absorb or unstable.

The stability of hydrogels ensures the maintenance of their structure and function in the biological environment, while the bioavailability reflects the efficiency with which they deliver the active substance to the body. Optimizing these two characteristics is essential for medical applications such as controlled drug delivery, regenerative therapy, and intelligent treatment systems (Table 4).

### 3.4. Advantages and Limitations of Hydrogels

The first major advantage of hydrogels is their high water content, which results in a soft, elastic structure similar to that of living tissues. This makes them ideal for applications that require a moist environment, such as wound dressings or contact lenses. Hydrogels are also biocompatible, which means they can enter the human body without causing significant immune reactions. They are permeable to oxygen and nutrients, which aids in the biological exchanges required for cell regeneration [99].

The physical properties of hydrogels, such as porosity, elasticity, and stiffness, can be controlled by the polymer used and the degree of crosslinking, allowing them to be tailored to various applications. In addition, there are stimuli-responsive (or “smart”) hydrogels, which can change their properties in response to external factors such as temperature, pH, or light. These are extremely useful in modern controlled drug delivery systems [104].

Last but not least, hydrogels are extremely versatile, being used in regenerative medicine, tissue engineering, agriculture (for water retention in soil), the cosmetics industry, and the production of biological sensors.

Despite their benefits, hydrogels do have some disadvantages. First, their mechanical strength is low, making them fragile and limited in applications that require structural stability. Also, many hydrogels undergo dehydration or chemical degradation over time, thus losing their effectiveness.

Another issue is the sterilization process, which can damage the polymer network using traditional methods like autoclaving or radiation. Furthermore, controlling the release of active substances from the hydrogel can be difficult at times, resulting in an uneven or too rapid release.

Another important consideration is the cost of producing complex hydrogels, as well as the risk of residual toxicity from monomers or crosslinkers that are not completely removed during synthesis.

Although hydrogels are novel materials with enormous potential due to their biocompatibility, water absorption capacity, and versatility, their widespread use is hampered by mechanical fragility, long-term instability, and sterilization issues.

Recent research has focused on the development of hybrid or nanocomposite hydrogels, which combine natural and synthetic polymers to create stronger, more durable, and safer materials. With these advancements, hydrogels may become indispensable in future medicine and a wide range of industrial applications [114].

Table 5 shows the properties of hydrogels that provide advantages and limitations to their applicability.

### 3.5. Applicability of Hydrogels in the Prevention of Metabolic Diseases

Metabolic diseases are a complex category of conditions characterized by dysregulation of basic biochemical processes such as glucose, lipid, and protein metabolism. Traditional therapeutic approaches are limited in terms of drug stability, biodistribution, and side effects when administered systemically. In this context, hydrogels have emerged as novel solutions due to their ability to control the release of active substances and selectively interact with the physiological environment [115].

Hydrogels are not merely theoretical concepts. Formulas are already in clinical trials and available on the market (chitosan supplements, satiety capsules with natural hydrogels). Hydrogels are being explored in a number of applications for metabolic diseases, particularly for treatment of components of the metabolic syndrome (diabetes, obesity) and for complications (e.g., fatty-liver, diabetic wounds) [116,117].

Hydrogels can be applied subcutaneously, intragastrically, via microneedles, or as topical dressings due to their flexible delivery methods, which enables site-specific treatment of wound problems, diabetes, obesity, and NAFLD. When combined, these characteristics enable hydrogels to offer tissue-targeted, patient-friendly, and regulated treatment for a wide range of metabolic diseases [118].

Smart hydrogels react to pH, temperature, or enzymes, allowing for controlled oral delivery and improved absorption of bioactive ingredients in the gastrointestinal tract through diffusion, swelling, and gradual erosion. Hydrogels can also transport vitamins, antioxidants, probiotics, and bioactive peptides to protect against inflammation and oxidative stress [119].

#### 3.5.1. Diabetes, Control of Glucose and Lipid Absorption

The most common application of hydrogels is the treatment of diabetes. Glucose-sensitive hydrogels, based on polymers such as polyvinyl alcohol (PVA) or chitosan modified with phenylboronate groups, can regulate insulin release in response to blood glucose levels [115].

In parallel, 3D bioprinted hydrogels made of alginate and PEG create biocompatible environments for encapsulating pancreatic β cells, protecting them from the immune system and facilitating cell transplantation [120].

Hydrogels can prevent type 2 diabetes by forming physical barriers in the intestine and reducing postprandial glycemic peaks [121]. Can be used for the slow and stable release of pharmacological substances with a preventive role in metabolic syndrome (e.g., metformin, GLP-1 agonists, and natural polyphenols). Modified GLP 1, insulin cell derived extracellular vesicles, and telmisartan are all delivered by an injectable glucose responsive alginate/3 aminophenyl boronic acid hydrogel (Diabogel); it lowers blood glucose, maintains body weight, and significantly reduces hepatic and renal toxicities while causing pancreatic macrophages to adopt an anti-inflammatory phenotype [122]. Similarly to daily injections, an injectable depot that adjusts hydrogel characteristics to retain long-acting GLP 1 receptor agonists offers months of exposure (about 42 days in rats) and achieves weight and glucose management, reducing treatment burden and enhancing adherence [123]. Also an alginate graphene oxide hydrogel supports encapsulated pancreatic β cells, enhancing viability, proliferation and insulin secretion while offering a compact, low swelling matrix suitable for in vitro diabetes treatment studies [124].

Biopolymer-based hydrogels (e.g., pectin, alginate, chitosan, whey proteins) can encapsulate fats or sugars, reducing their bioavailability and impact on blood glucose and serum lipids. They can mimic the texture of conventional foods while reducing calori intake [125]. Also, for diabetic wound healing, a dual-responsive hydrogel (PVA/CS-BA) releases insulin and celecoxib in response to high glucose and MMP-9 levels, accelerates wound closure, modulates inflammation and promotes tissue regeneration in diabetic skin-defect models [126].

#### 3.5.2. Dyslipidemia and Atherosclerosis

Hydrogels hold promise for the controlled delivery of therapeutic agents such as statins or nucleic acid molecules (e.g., siRNA) in the treatment of dyslipidemias [127]. These systems enable the gradual and localized release of active substances, keeping plasma concentrations within the optimal therapeutic range while lowering the risk of systemic side effects [128,129].

Hydrogels based on natural biopolymers, such as chitosan and alginate, have shown excellent biocompatibility and the ability to form stable three-dimensional structures that can incorporate and protect active agents from premature degradation [130]. In the case of statins, the use of these hydrogels helps to reduce the liver toxicity associated with traditional oral administration by avoiding the first-pass effect in the liver and directing the release to the affected vascular tissue.

Furthermore, the combination of targeted delivery technologies enables selective vascular targeting, which may result in superior therapeutic efficacy in reducing atherosclerotic plaques and improving overall lipid profile. Similarly, controlled siRNA delivery via hydrogels provides the opportunity to regulate the expression of genes involved in lipid metabolism, opening up new avenues for personalized therapies with minimal side effects [131].

#### 3.5.3. Obesity and Metabolic Syndrome

A rapidly growing class of non-pharmacologic obesity treatments, hydrogel-based devices take advantage of water-absorbing polymers’ capacity to fill gastric space, postpone nutrition passage, and elicit satiety signals without exposing users to systemic drugs. Super-absorbent hydrogels quickly expand in the stomach’s acidic environment after consumption, creating a three-dimensional gel that inhibits peristalsis and decreases the amount of space available for food, improving early satiety and reducing caloric intake. In order to maintain volume during gastric emptying, dual-network topologies that combine a stiff backbone with a highly elastic secondary network or polymer networks that can absorb 80–100 × their dry weight (such as Plenity^®^/Gelesis100, Boston, MA, USA) are responsible for the swelling [132]. Also, adipose tissue inflammation and pancreatic dysfunction are major contributors to obesity and diabetes. Biomimetic hydrogels can support pancreatic cell transplantation and regenerative therapies and cellulose-based superabsorbent hydrogels can mimic raw vegetable elasticity, preserve gut tissue integrity and act as non-systemic mechanotherapeutics that aid weight-management, with a cleared product already marketed in the US/Europe [133]. All things considered, hydrogel systems offer a flexible, mechanically based method of managing obesity that makes use of volume-occupying qualities, good safety, and the ability to be integrated with lifestyle changes, making them a viable supplement or substitute for current treatment options.

#### 3.5.4. Non-Alcoholic Fatty Liver Disease (NAFLD)

In order to treat non-alcoholic fatty liver disease (NAFLD), hydrogel technologies are being modified to carry medications straight to the liver, fix metabolic pathways, and provide scaffolds for tissue healing. The same design ideas are being used to hydrogel matrices, despite the fact that the majority of contemporary nanocarrier research for NAFLD concentrate on lipid nano-emulsions or polymeric micelles. For instance, oral bioavailability of flavonoids for early treatment of nonalcoholic fatty liver disease (NAFLD) is improved by stiff-soft hybrid biomimetic nano-emulsions; incorporating these compounds into injectable or intraperitoneal hydrogels may further extend hepatic exposure while restricting systemic distribution [134]. To enhance drug bioavailability and target metabolic dysfunction–associated fatty liver disease (MAFLD), a transdermal hydrogel patch (Vera@CLCMP) was developed. Its hierarchically porous structure allows continuous verapamil release and improved skin penetration without altering the drug’s chemistry. In diet-induced obese mice, the patch showed noninvasive, liver-targeted effects by restoring autophagy, suppressing the TXNIP/NLRP3 inflammasome, reducing hepatic lipid accumulation, and improving insulin sensitivity [135]. In another study, a scalable in vitro platform that preserves the natural biochemical cues of the diseased organ has been created by encapsulating human multicellular NAFLD microtissues in a hydrogel made from liver extracellular matrix. These ECM hydrogels are generally used as research tools, but they also allow for quick screening of anti-steatotic drugs and may be modified for in vivo localized administration of medicinal medicines [136].

In conclusion, hydrogels are being used to treat nonalcoholic fatty liver disease (NAFLD) by delivering liver-targeted, metabolic-modulating medications like verapamil over an extended period of time, offering biomimetic scaffolds that improve hepatocyte function, and acting as flexible platforms for multimodal therapies that tackle the intricate pathophysiology of fatty liver disease.

These examples illustrate how hydrogel platforms can prevent or mitigate metabolic disorders by delivering therapeutics, protecting functional cells, and addressing complications such as chronic wounds.

To better illustrate the role of hydrogels, Table 6 shows their mechanism of action and applications by hydrogel type.

Future directions include smart nanohydrogels that can deliver nutrients or drugs to the target in a personalized manner.

## 4. Discussion

Through a comparative view of liposomal nanosystems and hydrogels for the prevention and treatment of metabolic diseases (diabetes, obesity, non-alcoholic steatohepatitis/non-alcoholic fatty liver disease, etc.), both platforms appear promising but address different challenges (Table 7). Therefore:

- Liposomes and related lipid nanoparticles are effective at systemic delivery, improving oral/IV bioavailability, targeting, and protecting fragile payloads like peptides, small molecules, and nucleic acids. They are widely used in nanomedicine and are being adapted to treat metabolic diseases (insulin/peptide delivery, hepatic/adipose targeting, improved bioactive solubility).
- Hydrogels can be used as local depots, tissue scaffolds, or transmucosal/implantable systems for sustained local release, cell or microbiome modulation, or injection into adipose tissue or the pancreas microenvironment. They are particularly appealing where local control, mechanical support, or extended release is required.

Although much of the current research emphasizes preclinical and proof-of-concept studies, the clinical translation of liposomal and hydrogel-based formulations for metabolic diseases remains limited. On the liposomal side, several products—such as Doxil^®^ (Horsham Township, PA, USA) and Arikayce^®^ (Bridgewater, NJ, USA)—have already gained FDA approval for oncology and infectious disease indications, demonstrating the maturity and safety of liposomal technology platforms [137,138,139]. However, none of these formulations currently target metabolic disorders such as type 2 diabetes (T2D), obesity, or dyslipidemia. Recent reviews highlight that liposomal formulations designed for metabolic applications—such as the encapsulation of insulin sensitizers [54] lipid-lowering agents [57,140], or anti-obesity compounds [63]—remain almost entirely within the preclinical research stage, with very few candidates advancing toward clinical evaluation. Although liposomal technology has demonstrated significant potential in improving bioavailability, stability, and targeted delivery of pharmacologically active compounds, its translation into the metabolic and nutritional disease domain has been limited compared to its established success in oncology and infectious disease therapies. For instance, a recent comprehensive review emphasized that studies involving liposomal encapsulation of bioactive phytochemicals targeting obesity-related pathways remain predominantly at the experimental level, with scarce evidence of clinical validation [12]. Similarly, broader evaluations of liposomal systems have noted persistent challenges related to formulation stability, scalability, and regulatory standardization, which collectively hinder their clinical translation [141,142]. Moreover, recent analyses of liposomal insulin and oral peptide delivery platforms reaffirm that, despite decades of preclinical progress, these systems remain under investigation with limited human trial data available [54]. These findings underscore the need for improved translational strategies—encompassing formulation optimization, long-term stability assessment, and scalable manufacturing—to facilitate the advancement of liposomal nanosystems from preclinical promise to clinical implementation in metabolic disorders.

In parallel with liposomal systems, hydrogel-based formulations have gained attention for controlled drug delivery in metabolic disorders. Composed of natural or synthetic polymers—such as alginate, chitosan, hyaluronic acid, and polyethylene glycol—hydrogels have been explored as carriers for insulin, incretin mimetics, and bioactive plant-derived compounds due to their biocompatibility, tunable degradation, and sustained-release capacity [143,144]. For example, an injectable supramolecular hydrogel depot for a GLP-1 receptor agonist (GLP-1 RA) achieved sustained release for ~42 days in rat models of T2D, corresponding to roughly four months of therapeutic exposure in humans, with glycemic and weight control comparable to daily dosing [123]. Despite these promising results, most hydrogel platforms for metabolic therapy remain in preclinical or early proof-of-concept stages. Only a few insulin formulations—primarily transdermal or oral—have reached preliminary clinical assessment, facing challenges such as enzymatic degradation, mucosal permeability, and batch reproducibility [144,145,146,147]. Advanced multifunctional hydrogels capable of responding to physiological stimuli, such as glucose or pH fluctuations, are under investigation to enable on-demand drug release [143], but translation to clinical use requires addressing scalability, regulatory validation, and long-term safety [148].

Among the various hydrogel classes, semi-synthetic and natural biopolymer hydrogels currently show the greatest clinical readiness for metabolic disorder management. Chitosan-, alginate-, and gelatin-based hydrogels are already approved for several biomedical uses due to their safety, biodegradability, and mucosal adhesion, and have advanced into clinical evaluation for oral and injectable metabolic applications [73]. Semi-synthetic derivatives such as methacryloyl-modified gelatin (GelMA) and acrylated hyaluronic acid (AcHyA) offer improved mechanical strength and tunable degradation, supporting their use as injectable depots for pancreatic cell transplantation or adipose tissue remodeling [78,149,150]. For example, Plenity^®^ (Gelesis100)—an FDA- and EMA-approved hydrogel for obesity management in 2019—was based on superabsorbent cellulose–citric acid crosslinked hydrogel technology [151]. Due to some severe adverse reactions, which have affected safety and tolerability, it was discontinued in 2022 [152].

In contrast, *Diabogel*—a glucose-responsive alginate/3-aminophenylboronic acid injectable hydrogel—has shown promising preclinical results for long-term glycemic control and weight management [122], but it is not (yet) approved by major regulatory authorities. These examples illustrate the translational readiness of natural and semi-synthetic hydrogels in the metabolic disease field. Collectively, hydrogel systems for diabetes and NAFLD remain at the preclinical or early clinical stage, reflecting ongoing translational development.

Notably, several hydrogel-based formulations have already achieved regulatory approval, demonstrating their translational maturity. Examples include Regranex^®^ (becaplermin gel, PEG-based) for diabetic ulcers [153], Restylane^®^ and Juvéderm^®^ (hyaluronic acid-based injectable hydrogels) for soft-tissue restoration [154,155], and Seprafilm^®^ (hyaluronic acid/carboxymethylcellulose barrier film) used to prevent postoperative adhesions [156]. These clinically approved systems highlight the established safety, biocompatibility, and regulatory acceptance of hydrogel technologies, supporting their potential adaptation for metabolic disease therapies. In contrast, fully synthetic hydrogels (e.g., PVA, PEG, or PNIPAM) provide superior mechanical and chemical stability but have slower regulatory progression due to limited biodegradability and potential cytotoxic residues [73]. Overall, naturally derived or semi-synthetic hydrogels represent the most clinically advanced and commercially viable materials for developing safe, biocompatible metabolic-disease therapies.

Quantitative data from studies in metabolic disease models indicate that liposomal carriers and hydrogel systems differ meaningfully in release kinetics and effective bioavailability, and that their combination (liposome-in-hydrogel) often gives the most favorable profile. Fenofibrate liposomes showed a 5.13-fold increase in relative bioavailability versus micronized capsules [8]. Insulin liposomes embedded in alginate hydrogels (AINS-Lip-Gel) exhibited a relative bioavailability of 9.82 ± 0.56% compared to subcutaneous injection [5], while hydrogel-based insulin depots (Diabogel) achieved sustained glucose control for up to 42 days in animal models [122,123]. These data emphasize the complementary release kinetics and systemic exposure achieved by liposomal and hydrogel carriers.

For oral insulin delivery, Wu et al. (2023) reported that an alginate hydrogel embedding arginine-insulin complexes contained in liposomes (AINS-Lip-Gel) produced ≈6-fold greater ex-vivo intestinal permeation than free insulin and ≈3-fold greater permeation than AINS-Lip alone; the hydrogel also retarded the early release from liposomes (only ~30% early release) and produced a sustained in vivo hypoglycemic effect compared with controls [20]. In simulated gastric conditions (pH 1.2) liposomes entrapped in hydrogels released substantially less insulin (≈10% over 3 h) than liposomes alone (≈40% over 3 h), showing that the hydrogel matrix reduces burst release and protects the vesicles in harsh gastric conditions [20,157].

For hepatoprotective and metabolic bioactives, liposomal encapsulation has been repeatedly shown to improve systemic exposure: silibinin-loaded liposomes increased absorption and therapeutic efficacy in NAFLD models compared with non-encapsulated silibinin, whereas hydrogels or hydrogel composites (e.g., mucoadhesive or depot formulations) primarily provide localized, sustained release and improved retention at the delivery site—an advantage for local adipose or pancreatic depots but less useful for achieving rapid systemic exposure [31,158].

Overall, current evidence highlights three key practical considerations for applications in metabolic diseases: (1) liposomes substantially improve solubility and systemic bioavailability of poorly soluble nutraceuticals and drugs (e.g., silybin, resveratrol, curcumin) and can increase absorption in vivo; (2) hydrogels excel at reducing initial burst release and creating long-acting local depots (useful for injectable adipose or pancreatic microenvironments); and (3) liposome-in-hydrogel composites combine the benefits of both—protection of sensitive compounds (by liposomes) plus suppression of early burst and increased mucosal retention (by hydrogels)—leading to both improved intestinal uptake and prolonged release in metabolic disease models [20,31,158]. These quantitative findings justify considering liposome-in-hydrogel formats when the target is oral or mucosal delivery of peptides (e.g., insulin) or when both systemic bioavailability and sustained local action are required in metabolic disease interventions.

Building on the complementary strengths of liposomes and hydrogels, hybrid liposome–hydrogel systems represent a logical and practical next step in the development of delivery platforms for metabolic disease therapy. These composite carriers combine the high encapsulation efficiency and cellular uptake of liposomes with the mechanical stability and sustained-release behavior of hydrogels, thereby addressing key limitations of each single system [20,31]. In metabolic disease contexts, e.g., diabetes, obesity, and non-alcoholic fatty liver disease (NAFLD), such hybrid systems can offer dual benefits: systemic bioavailability through liposomal encapsulation and localized, prolonged release provided by the hydrogel matrix. Nevertheless, major formulation challenges remain, including maintaining the structural integrity of liposomes within hydrophilic polymer matrices, preventing premature leakage of bioactives during gelation or storage, and achieving reproducible large-scale manufacturing with minimal batch variability [158]. Furthermore, ensuring the stability of bioactive compounds under physiological conditions and developing sterilization methods that preserve both lipid vesicle integrity and hydrogel network properties are critical to achieving translational success.

Translating these advanced delivery systems into clinical practice requires navigating complex regulatory pathways, which differ substantially depending on whether the formulation is intended as a nutraceutical or a pharmaceutical. For pharmaceutical applications, both liposomal and hydrogel systems are regarded as advanced drug-delivery formulations that must comply with stringent pharmacokinetic, safety, and stability evaluations defined by agencies such as the EMA and FDA [26, 101,159,160,161]. Liposomal drugs are classified under the “nanomedicine” category and require detailed characterization of size, lamellarity, encapsulation efficiency, and release kinetics [162]. In contrast, hydrogels used as medical devices or combination products are regulated according to their polymer composition and functional role [73]. For nutraceutical applications, regulatory demands are less rigorous: liposomal or hydrogel systems delivering bioactive nutrients (vitamins, polyphenols, or probiotics) are primarily evaluated for compositional safety and bioavailability enhancement rather than full clinical pharmacology [3]. However, once claims move from “supporting health” to “treating or preventing disease,” the product must transition into the pharmaceutical category. Thus, the boundary between nutraceutical and pharmaceutical formulations is largely defined by intended clinical claims and demonstrated therapeutic outcomes.

In addition to formulation and regulatory considerations, the choice of administration route plays a critical role in achieving therapeutic efficacy, depending on the target tissue and the physicochemical properties of the bioactive compound. Liposomal systems are most promising for oral and injectable delivery in metabolic diseases, owing to their capacity to improve absorption of poorly soluble drugs and protect peptides such as insulin from degradation [20]. PEGylated and bile-salt-modified liposomes have shown enhanced intestinal permeability and systemic bioavailability of antidiabetic and hepatoprotective compounds [8,161]. Conversely, hydrogels demonstrate greater potential for localized or transmucosal administration—such as injectable depots for adipose tissue modulation or glucose-responsive insulin release systems—and for oral superabsorbent hydrogels that modulate intestinal absorption and improve metabolic parameters [149,150]. Emerging nasal and transdermal hydrogel systems also provide non-invasive alternatives for delivering hormones or peptides while maintaining sustained systemic levels. Therefore, for systemic bioavailability, liposomal formulations are advantageous, whereas for local or sustained metabolic modulation, hydrogel and hybrid depot systems are more appropriate.

Altogether, these observations underscore that liposomal, hydrogel, and hybrid systems each offer distinct advantages, and their rational selection should be guided by the therapeutic goal, desired release profile, and target site in metabolic disease interventions.

## 5. Conclusions and Perspectives

Both liposomal nanosystems and hydrogels have significant potential for preventing and treating nutritional diseases. The choice between the two is determined by the nature of the active compound, the preferred route of administration, and the specificity of the target condition.

Overall, liposomal systems are more advanced clinically but underutilized in metabolic diseases, whereas hydrogel depots, though earlier in development, show strong potential for long-acting, localized therapy. Interdisciplinary strategies integrating formulation optimization, stability assessment, and scalable manufacturing are essential to translate preclinical promise into clinical applications.

The future will most likely bring hybrid systems that combine the benefits of both technologies, opening up new avenues for personalized therapeutic nutrition.

## 6. Future Challenges and Prospects

In future research, for the biomedical field, hydrogels can be considered from the following three aspects of in-depth research: first, improve the mechanical properties to meet the demand of its application in tissue engineering; second, in-depth compound modification research with other materials to develop better properties (such as rapid degradation, biocompatibility, etc.) for medical applications; third, combined with new molding means [31], such as 3D printing technology, to prepare a personalized hydrogel.

For liposomal nanosystems, future research should focus on improving clinical translation for metabolic disorders. Despite decades of preclinical work showing improved bioavailability, targeted delivery, and protection of fragile compounds [12,54,57], few formulations have reached clinical evaluation. Moving forward, efforts should aim at optimizing formulations for long-term stability, scalable manufacturing, and regulatory compliance [141,142], as well as developing strategies for oral or mucosal delivery of peptides such as insulin [20]. Integration of novel targeting ligands, PEGylation strategies, or bile-salt modifications could further enhance systemic bioavailability and organ-specific delivery [8,163].

For hydrogel systems, future challenges involve refining material properties and administration strategies. Semi-synthetic and natural biopolymer hydrogels (e.g., chitosan, alginate, gelatin, GelMA, AcHyA) already show strong clinical readiness [149,150,156], but improvements in mechanical strength, tunable degradation, and stimulus-responsiveness are needed to meet the demands of tissue engineering, local drug depots, or glucose-responsive systems [123,143]. Additionally, new fabrication technologies, such as 3D printing, could enable personalized hydrogel-based therapies with precise geometry and payload distribution [86]. Addressing enzymatic degradation, mucosal permeability, and reproducibility remains critical to achieving clinical success [144,145].

Hybrid liposome–hydrogel systems represent a particularly promising direction, combining the systemic bioavailability advantages of liposomes with the sustained, localized release of hydrogels [20,31,158]. Future research should focus on overcoming formulation challenges, such as maintaining liposome integrity within hydrophilic polymer matrices, preventing premature leakage of bioactive, and developing sterilization methods compatible with both components. Scalable, reproducible manufacturing methods and long-term stability assessment will be crucial to translate these hybrid systems from preclinical models to clinical applications [20,158].

Finally, regulatory and administration considerations will continue to shape development strategies. Liposomal and hydrogel platforms must comply with distinct regulatory pathways depending on their classification as nutraceuticals or pharmaceuticals [26,156,159]. Rational selection of the administration route—oral, injectable, transmucosal, or non-invasive alternatives—should align with the bioactive properties and therapeutic goals, balancing systemic bioavailability with local, sustained modulation [20,149,150].

Overall, the future of metabolic-disease therapy will likely involve a combination of advanced liposomal, hydrogel, and hybrid platforms, developed through interdisciplinary strategies integrating material science, pharmacology, and clinical translation [20,31,141]. These approaches offer the potential for personalized, long-acting, and targeted nutritional or pharmacological interventions.

## Figures and Tables

**Table 1:** Advantages and limitations of liposomal nanosystems relevant for metabolic diseases prevention and treatment.

Type	Key Features	Advantages	Limitations	References
Conventional liposomes	Phospholipid bilayer vesicles with aqueous core	Biocompatible and versatile (carry both hydrophilic and lipophilic drugs) Protects drugs from enzymatic degradation	Susceptible to oxidation, hydrolysis, fusion Rapid clearance by the reticuloendothelial system (RES) Low oral bioavailability	[54,55,56]
PEGylated stealth liposomes	Surface grafted with polyethylene glycol (PEG) chains	Increased colloidal stability Prolonged circulation and half-life Reduced opsonization and RES clearance Improved pharmacokinetics of hydrophilic/labile drugs	Accelerated blood clearance (ABC) effect upon repeated dosing PEG layer can hinder cellular uptake Regulatory and immunogenicity concerns	[57,58,59]
Stimuli-responsive liposomes	Engineered to release payload in response to pH, redox, enzymes, or external stimuli (temperature, ultrasound, light)	Triggered, controlled release at target sites Higher local drug bioavailability Reduced systemic toxicity Potential for precision therapy	Complex formulations, harder to manufacture at scale Trigger efficiency can vary in vivo Stability during storage may be lower than conventional liposomes	[60,61]
Hybrid liposomal carriers	Liposomes integrated with polymers, metals, or silica-based nanostructures	Enhanced structural stability Sustained and controlled release profiles Higher loading efficiency for hydrophobic drugs Enable multimodal therapy (drug + imaging, externally triggered release)	Complex and costly synthesis Regulatory and translational challenges Long-term safety of hybrid materials remains uncertain	[62,63]

**Table 2:** Applicability of liposomal nanosystems.

Metabolic Disease	Therapeutic Agent/Drug Class	Liposomal Formulation	Advantage
**Type 1 and Type 2 Diabetes**	Insulin, Insulin analogs	Modified-release liposomes; Propanoic acid-functionalized chitosan-decorated liposomes	Enhanced oral bioavailability; prolonged hypoglycemic effect; protection from enzymatic degradation
	Sulfonylureas (Glibenclamide)	Conventional liposomal encapsulation	Improved stability; sustained drug release; reduced GI side effects
**Dyslipidemia/Atherosclerosis**	Statins (Atorvastatin, Simvastatin)	Liposomes with cationic gemini surfactants; Lyophilized liposomes	Enhanced drug retention and stability; controlled release; optimized pharmacokinetics
	Fibrates (Fenofibrate)	Liposomes containing bile salts	Increased solubility and bioavailability; reduced systemic toxicity
**Obesity/Metabolic Syndrome**	Thyroid hormone	Glucagon-modified liposomes	Targeted delivery to adipose tissue; improved fat metabolism; anti-obesity effect
	Peptide therapeutics (PDBSN)	Liposome-encapsulated peptide	Ameliorates high-fat-diet-induced obesity; restores metabolic homeostasis
**Non-Alcoholic Fatty Liver Disease (NAFLD)**	Lipid-modulating agents/antioxidants	Lipid-based liposomes targeting hepatocytes	Reduces oxidative stress, inflammation; improves hepatic lipid metabolism
**Enzyme Replacement Therapy**	Metabolic enzymes	Stimuli-responsive liposomes; oral or parenteral liposomes	Protects enzymes from degradation; targeted release; enhanced therapeutic efficacy

**Table 3:** The main types of hydrogels and their medical functions.

Field of Use	Examples of Hydrogels	Main Functions
**Medical**	Hydrogel dressings, contact lenses, injectable hydrogels	Tissue hydration and protection Wound healing promotion Support for controlled drug release Creation of a tissue-like environment for cells
**Pharmaceutical/Biotechnological**	pH or temperature sensitive hydrogels (e.g., PNIPAAm)	Intelligent and controlled drug release Transport of proteins, enzymes or DNA Sensors and biosensors
**Cosmetic**	Hydrogel facial masks, moisturizing patches	Skin hydration Delivery of active substances (vitamins, collagen) Comfort and cooling effect

**Table 4:** Bioavailability of hydrogels depending on the route of administration.

Route of Administration	The Role of the Hydrogel	Impact on Bioavailability	Application Examples
**Oral**	Protects the active substance from the gastric environment and enzymes; controlled release in the intestine	Variable bioavailability—good for sensitive peptides/proteins, but limited by intestinal absorption	Insulin, peptides, probiotics
**Transdermal**	Moisturizes the skin, increases skin permeability, allows prolonged diffusion	Significant increase in local and systemic bioavailability, especially for small and lipophilic molecules	Analgesics, hormones (e.g., estrogen), nicotine
**Injectable (subcutaneous/intramuscular)**	Forms a local depot, sustained and prolonged release	Very high and stable bioavailability, avoids plasma fluctuations	Vaccines, growth factors, antibiotics
**Ophthalmic**	Increases contact time with the ocular surface, protects from tear drainage	Improved ocular bioavailability (usually several times higher than simple solutions)	Glaucoma treatment, inflammation, infections
**Nasal/Pulmonary**	Good adhesion to mucous membranes, protects the active substance from degradation	Rapid bioavailability and sometimes comparable to injectable bioavailability	Vaccines, peptides, asthma treatments

**Table 5:** Advantages and limitations of hydrogels.

Property	Advantages	Limitations
**Biocompatibility**	Most are well tolerated by the body and appropriate for medical applications.	Some synthetic hydrogels may trigger allergic or inflammatory reactions
**Water Absorption**	Can retain large amounts of water and mimic natural tissues.	Excess water can reduce mechanical strength
**Permeability**	Allows the diffusion of oxygen and nutrients.	Uncontrolled diffusion can affect uniform drug release
**Flexibility**	Soft, comfortable and tissue-like.	Low mechanical strength, prone to breaking or deformation.
**Controlled Release**	Can serve as drug delivery systems.	If the structure is not optimized, release can be uneven.
**Self-Repair**	Some hydrogels can recover from deformation.	This property is not common to all types
**Applicability**	Medicine, Cosmetics, Tissue Engineering	Processing and shaping are complicated and costly.
**Stability**	-	Sensitive to temperature, pH, and enzymes; rapid degradation in the case of natural hydrogels
**Sterilization**	-	Can be difficult without loss of properties

**Table 6:** Table comparing types of hydrogels.

Hydrogel Type	Examples	Mechanism of Action	Application in Prevention of Metabolic Diseases
**Natural**	Alginate, pectin, chitosan, gelatin, whey protein	Biocompatible, biodegradable; forms gels in aqueous media; can encapsulate nutrients or drugs	Encapsulation of polyphenols and probiotics Reduction in glucose and lipid absorption Formulation of functional foods with low glycemic index
**Semi-synthetic**	Cellulose derivatives, modified collagen, derived chitosan	Adjustable properties (porosity, electrical charge); combines the advantages of nature and synthesis	Controlled release systems for antioxidants or vitamin D Intestinal barriers against rapid carbohydrate absorption Increased bioavailability of protective nutrients
**Synthetic**	Polyethylene glycol (PEG), polyacrylamide, polyvinyl alcohol (PVA)	Allows precise control of porosity and release; high stability, but less biodegradability	Delivery of diabetes/obesity drugs (e.g., metformin, GLP-1 agonists) Support for regenerative therapies (pancreatic β cells) Design of sustained-release anti-obesity materials
**Nanohydrogels**	Nanoparticle hydrogels (e.g., chitosan or PEG nanoparticles)	Large surface area, increased cellular absorption, protection against gastric degradation	Targeted delivery of polyphenols, resveratrol, curcumin Modulation of gut microbiota Increasing the efficiency of metabolic therapies

**Table 7:** Head-to-head comparison (practical points).

Dimension	Liposomal Nanosystems	Hydrogels
**Primary use case**	Systemic delivery, targeted organ/cell uptake, oral/IV peptide or small-molecule delivery	Local depots, injectable sustained release, scaffolds for cell delivery, wound/tissue modulation
**Payload types**	Hydrophilic drugs (core), hydrophobic (bilayer), nucleic acids (with modification), peptides/proteins	Small molecules, proteins, cells, living probiotics; can embed nanoparticles (including liposomes)
**Control of release**	Tunable via lipid composition, PEGylation, surface ligands; often burst + controlled phases	Wide—diffusion, degradation, stimulus-responsive chemistries (pH, enzymes, temperature) enable long sustained release
**Targeting ability**	High—ligand targeting, size/surface tuning for liver/adipose uptake; favors systemic targeting	Local by design; can be functionalized to attract cells or respond to local cues
**Barrier crossing/bioavailability**	Good for IV, improving oral bioavailability is challenging but progressing (enteric coatings, mucoadhesive surface)	Good for local tissue retention; not for deep systemic targeting unless combined with carriers
**Biocompatibility and clearance**	Generally biocompatible; lipid composition affects clearance and immune recognition (RES uptake). Scale-up and reproducibility manageable (some approved liposomal drugs exist)	Many are biocompatible (natural/synthetic options); mechanical strength and batch-to-batch variability can be issues. Several hydrogel products already in clinic.
**Manufacturing and regulatory**	Matureer for lipid formulations; regulatory path available but needs careful characterization (size, encapsulation, stability)	Some hydrogel therapies are FDA/EMA approved (mainly device/implant or wound products). Drug-eluting hydrogel combinations require combined device + drug regulatory strategies.
**Best when**	You need targeted systemic delivery (e.g., hepatic delivery of small molecules or nucleic acids; protect peptide drugs)	You want local, long-acting release (e.g., adipose microenvironment modulation, localized insulin depot, tissue engineering)
**Typical limitations**	Stability in biological fluids, opsonization, limited oral success for large biologics (though improving).	Mechanical weakness for load-bearing sites, potential burst release if poorly designed, sterilization challenges.

## Data Availability

No new data were created or analyzed in this study. Data sharing is not applicable to this article.
